# A Case of Ureter Herniation in the Petit Triangle

**DOI:** 10.5811/cpcem.49084

**Published:** 2026-03-29

**Authors:** Atsuhito Tanaka, Yuka Kamitani

**Affiliations:** * Tokyo Bay Urayasu Ichikawa Medical Center, Department of Emergency and Critical Care Medicine, Chiba, Japan; † Kobe City Medical Center General Hospital, Emergency Care Center/Emergency Department, Kobe, Japan

**Keywords:** ureter herniation, Petit triangle

## Abstract

**Case Presentation:**

An 88-year-old man was brought to our emergency department due to altered mental status and hemodynamic shock due to a urinary tract infection. Computed tomography showed an incarcerated ureter in the Petit triangle. Urology was consulted, and the hernia was reduced back into the retroperitoneal cavity.

**Discussion:**

Petit hernia is rare; moreover, there is no literature to our knowledge discussing the ureter as the herniated structure. Interventional radiology can be considered as a reductive option.

## CASE PRESENTATION

**Image 1:**
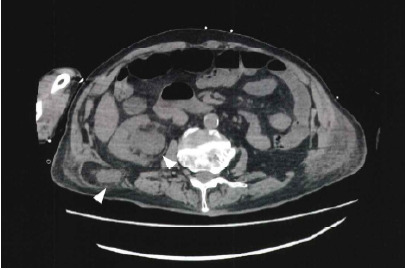
Computed tomography (axial view) showing the dilated renal pelvis and herniated ureter from the Petit triangle.

**Image 2:**
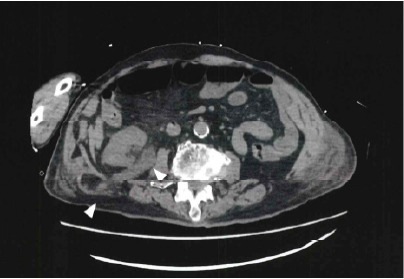
Computed tomography (axial view) caudal of Image 1, showing the dilated ureter and herniation.

**Image 3:**
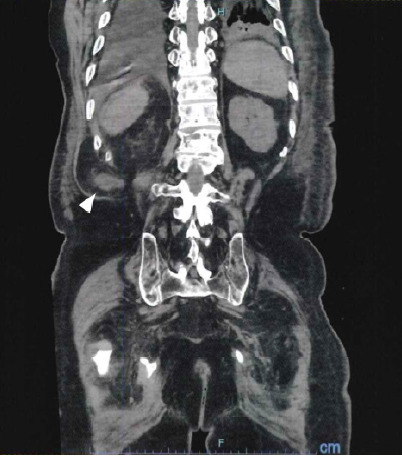
Computed tomography (coronal view) showing the herniated ureter through the Petit triangle (arrow).

An 88-year-old man was transferred to our emergency department with a decreased level of consciousness and elevated inflammatory markers. His past medical conditions included osteomyelitis and cerebral infarction. No history of surgery or trauma was noted. On arrival, he was in septic shock for which vasopressor infusion and antibiotics were administered. Blood tests revealed elevated creatinine, and point-of-care ultrasound demonstrated dilatation of the right renal pelvis. Computed tomography (CT) of the abdomen was performed to determine the etiology of altered mental status and possible post-renal obstruction. Abdominal CT revealed an incarcerated right ureter that had herniated through the Petit triangle in the inferior lumbar region, accompanied by right renal pelvic dilatation (Images 1–3). Urology was consulted, and the ureter was retrogradely reduced back into the retroperitoneal cavity via cystoscopy. Renal function improved after the intervention, with a decrease in serum creatinine from 2.57 milligrams per deciliter (mg/dL) to 1.83 mg/dL (reference range: 0.61–1.04 mg/dL). The patient recovered and was discharged on day 10.

*CPC-EM Capsule*

What do we already know about this clinical entity?

*Petit lumbar hernia is a rare type of lumbar hernia*.

What is the major impact of the image(s)?

*The images confirm a rare ureteral herniation into the Petit triangle while validating a successful endourological treatment option*.

How might this improve emergency medicine practice?

*Ureteral lumbar hernia should be on the differential for acute kidney injury and validates endourological reduction as a minimally invasive approach*.

## DISCUSSION

Petit hernia, a rare type of lumbar hernia, occurs through the space formed by the iliac crest, external oblique muscle, and latissimus dorsi muscle in the posterior abdomen, representing 1.5–2% of all hernias.^1^ Most reported cases discuss herniation of the colon or small bowel, often requiring surgical intervention. Ureteral hernias are uncommon clinical entities, making this specific combination unprecedented.^2^,^4^,^5^ Considering the patient’s age and clinical status, a less-invasive approach was chosen over surgical intervention. Zandrino et al described a percutaneous anterograde approach to resolve a lumbar hernia in the first case report of a hernia retrogradely reduced back into the retroperitoneal cavity via cystoscopy and guidewire manipulation. This suggests that minimally invasive radiological or endourological techniques could be a primary therapeutic option in such rare cases.
